# Key performance indicators at FIFA Women's World Cup in different playing surfaces

**DOI:** 10.1371/journal.pone.0241385

**Published:** 2020-10-23

**Authors:** Jorge Garcia-Unanue, Alvaro Fernandez-Luna, Pablo Burillo, Leonor Gallardo, Javier Sanchez-Sanchez, Samuel Manzano-Carrasco, Jose Luis Felipe

**Affiliations:** 1 IGOID Research Group, Universidad de Castilla-La Mancha, Toledo, Spain; 2 Faculty of Sport Sciences, Universidad Europea de Madrid, Villaviciosa de Odón, Spain; Universidade Federal de Juiz de Fora, BRAZIL

## Abstract

The aim of this study was to examine the key performance indicators of female professional soccer players during the 2011 and 2015 FIFA Women's World Cup, played on different surfaces (natural and artificial turf respectively). A total of 438 women from 24 national teams who participated at Canada 2015 (artificial turf) and 283 players from 16 national teams who played in Germany 2011 (natural grass) were selected for this study. The collected data were provided by OPTA Sports. Twenty-nine key performance indicators were included for analysis. The variables were calculated for the total sample and independently by positions (defense, midfielders and forwards) for matches on natural grass (2011) and artificial turf (2015). A Mann–Whitney U test was used out to identify differences between the sport surfaces. Moreover, a discriminant analysis was performed with the forced entry method to find the variables that better differentiated between the FIFA Women's World Cup 2011 (natural grass) and FIFA Women's World Cup 2015 (artificial turf). Key performance aspects were very similar between the two tournaments, but on natural grass, we observed a significantly higher number of total passes, successful dribbles, total tackles, successful tackles and interceptions. However, on artificial turf there were significantly higher percentages of success in total passes, and a higher number of fouls. This is an important factor for the choice of an elite competition surface because technical actions are crucial to the quality of the game and can influence the future behavior of spectators and fans.

## Introduction

Soccer is the most popular sport in the world. The main analysis in sports includes technical and tactical evaluation, movement analysis, feedback provision, norm development and modelling [1]. The complexity of soccer match performance can be reduced by using performance analysis techniques, which present the results in a systematic way, and integrate them into the coaching process [2]. According to Shafizadeh et al. [3], previous research has used different strategies in order to find the key performance indicators for success in soccer. Among the methods of performance measurement, one of the most analysed domains has been the quantification of technical actions and their success [4]. In this area, Garcia-Unanue et al. [5] showed that the physical performance of professional soccer players is influenced by game location, quality of the opposition and points required to maintain the category. On the other hand, Lago-Peñas [6] revealed that the physical and technical–tactical actions of soccer are influenced by situational variables that affect players at the behavioural level; the association between outcomes (notational analysis) and processes (spatiotemporal analyses) may also contribute to identify which patterns can be avoided or reinforced to increase the possibility of success [7]. Thus, the identification of players' actions and corresponding pitch location (as an example of indirect tactical information) that can lead to success in different competitive contexts may contribute to a better understanding of the key factors that influence performance in soccer [8]. Previous studies [9], have compared the performance of successful and unsuccessful teams during a FIFA (Fédération Internationale de Football Association) World Cup (1990 edition) and showed that converting possession into shots on goal was a key factor of successful teams. In this sense, ball possession has been reported as a critical indicator of team success [10], and Rein and Memmert [11] highlighted the connection between technical variables and the physical parameters of the elite soccer players. Thus, an examination of the technical performance that contributes to a teams' profile and quality/success would assist physical trainers, coaches and performance analysts to improve performance [12].

Another important aspect of research about soccer is the type of structure or tournament. Some of these studies have focused on international tournament soccer competitions, some at top-level cross border tournament competitions (e.g., UEFA (Union of European Football Associations) Champions League) and some on top-level domestic league soccer [13–15]. For example, Lago [16] at the 2006 FIFA World Cup, revealed that the technical and tactical indicators were relevant variables to explain the points obtained by the teams in the group stage of the competition. A similar result was found in the 2014 FIFA World Cup, which identified a positive effect of counter-attacks, ball possession, short passes and average passes completed per game in the likelihood of victory [17]. It was concluded [18] that ball possession did not influence the physical demands of the matches, even though it was related to the time spent in attacking sectors of the field. In the same competition [19] it was shown that seven of the teams that qualified for the quarter-final stage in the 2018 FIFA World Cup, used a combined tactical approach producing a better performance in the competition, no matter what the playing style of the team was.

Female soccer is becoming a very popular sport around the world, with an increasing number of players; however, despite this, we could not find any studies analyzing key performance indicators for women specifically, but only assessments of their physical capacities [20, 21]. In addition, in recent years, the use of artificial turf has grown exponentially. The constant evolution of these sports surfaces and the proliferation of artificial turf supply companies have encouraged the design of different types of artificial turfs (with a wide range of structural components), designed for soccer. One of the main advantages of artificial turf is its resistance to adverse weather conditions, therefore, it is used in first division championships faced with extreme-cold conditions, such as the Russian League. Several studies have demonstrated that artificial turf surfaces (especially in third-generation systems) do not have a negative impact on the performance of players and do not produce more injuries compared to natural grass surfaces [22–24]. In this regard, López-Fernandez et al. [25] analyzed the impact of these two surfaces on the movement profile of female soccer players during small-sided games. This study revealed that the women's performance (total distance covered, medium and peak speed and body load) was higher on artificial turf than natural grass, however, the natural grass surface showed the highest outcomes in the most intense small-sided games.

Currently, there are no studies on women-soccer key performance indicators on different surfaces. Nevertheless, we have found many studies about men’s performance during international soccer tournaments [14, 16], but authors never included the surface variable, because every professional competition is carried out always in natural grass pitches. For this reason, the objective of this research is double. On the one hand, to describe the key performance indicators of women-soccer in an international tournament, and on the other hand, to analyze if there are differences based on the playing surface, being our main hypotheses there will not meaningful differences in the key-performance indicators between the two types of surfaces.

Therefore, the aim of this study was to examine the key performance indicators of female professional soccer players during the 2011 and 2015 FIFA Women's World Cup, played on different surfaces (natural and artificial turf, respectively). We also took into account the players' position, rate of successful/unsuccessful actions, and also the comparison among the performance of players that were in both tournaments.

## Materials and methods

A total of 438 players from 24 national teams who participated at FIFA Women's World Cup Canada 2015 (played on artificial turf) and 283 players from 16 national teams who played at FIFA Women's World Cup Germany 2011 (played on natural grass) were selected for this study. Goalkeepers and soccer players who played less than 90 minutes were excluded, leaving a final sample of 313 different players of World Cup 2015 (median age: 28.69 ± 5.20), and 205 players of World Cup 2011 (median age: 26.72 ± 4.23). Data were obtained for each player per match.

The collected data were provided by OPTA Sports (London, UK), a private company dedicated to the performance assessment of teams in UEFA and FIFA tournaments (www.optasports.com). OPTA Sports prepared a budget based on the requested data, and Universidad Europea paid the fee to have access to the data of the competitions analyzed in this manuscript, under Grant 2015/UEM08. Thus, authors have not special privileges to access this data. OPTA’s software records the technical actions of players based on defined criteria. The software also allows the collection and immediate analysis of data gathered from the observation of matches. The accuracy of the Opta System has been verified by Liu et al. [26]. Reliability was assessed by the authors by coding five randomly selected players and their data being compared with that provided by Opta. The Kappa (K) values were ranged from 0.95 to 0.98.

A total of 29 key performance indicators were included for analysis (Table 1). The variables were calculated for the total sample, and independently by positions (defence, midfielders and forwards), and were divided into two groups: technical key performances indicators shown on natural grass matches (2011) and shown in artificial turf matches (2015). These indicators have previously been examined as valid indicators of match technical or skill performance in soccer [12, 17, 26–29].

**Table 1:** Key performance indicators analyzed in FIFA Women's World Cup 2011 and 2015.

Goals	Success rate of passes in the middle third	Success rate of dribbles
Shots	Passes in the final third	Tackles
Passes	Successful passes in the final third	Tackles won
Total successful passes	Success rate of passes in the final third	Success rate of tackles
Success rate of passes	Touches in own half	Clearances
Passes in the defensive third	Touches in the opposition half	Interceptions
Successful passes in the defensive third	Crosses	Fouls
Success rate of passes in the defensive third	Successful crosses	Yellow cards
Passes in the middle third	Dribbles	Red cards
Successful passes in the middle third	Successful dribbles	

The assumption of normality was analysed through the use of the Kolmogorov–Smirnov test. None of the variables followed the normal distribution. Following this exploratory analysis of the data, a descriptive analysis of the data was carried out. Then a Mann–Whitney U test was used out to identify differences between the sport surfaces. Effect sizes (ES) were also calculated following the recommendations for non-parametric data (TE = Z/√N, where Z = z-score of the Mann-Whitney U test and N = the total number of the sample). Lastly, a discriminant analysis was performed with the forced entry method to find the variables that better differentiated between the FIFA Women's World Cup 2011 (natural grass) and FIFA Women's World Cup 2015 (artificial turf). Average temperature of this tournaments was controlled (11 and 23 Celsius degrees in Canada and 10 and 20 Celsius degrees in Germany). The temperature was quite similar, and it did not influence the tournaments. The structural coefficients greater than 0.30 were considered relevant discrimination between groups [29, 30]. The statistical analyses were performed using SPSS software v.21.0 (IBM, Chicago, IL, USA), and statistical significance was set at p <0.05.

## Results

Table 2 shows the mean and standard deviations of the variables analysed at FIFA Women's World Cup 2011 (played in natural grass), and FIFA Women's World Cup 2015 (played in artificial turf). On natural grass, we observed a significantly higher number of total passes, passes in the defensive third, successful passes in the defensive third, touches in own half, total dribbles, successful dribbles, success rate of dribbles, total tackles, successful tackles and interceptions (ES from 0.09 to 0.43). However, on artificial turf, there were significantly higher percentages of success in total passes, passes in the middle third, passes in the final third, tackles and a higher number of fouls (ES from 0.11 to 0.27).

**Table 2:** Results of the variables analysed at the FIFA (Fédération Internationale de Football Association) Women's World Cup 2011 (natural turf) and 2015 (artificial turf).

	* *	*Total*		*Defenders*		*Midfielders*		*Strikers*	
		M	SD	M	SD	M	SD	M	SD
*Goals*	natural	0.11	0.22	0.02	0.08	0.10	0.22	0.26	0.28
	artificial	0.13	0.25	0.06	0.19	0.11	0.21	0.25	0.31
*Shots*	natural	1.11	1.21	0.28	0.42	1.32**	1.05	2.00	1.45
	artificial	0.98	1.09	0.35	0.50	1.01	1.07	1.81	1.15
*Passes*	natural	37.50*	11.26	39.23	9.01	41.25	12.47	28.88	7.16
	artificial	35.75	12.52	37.17	12.46	39.07	13.49	29.15	8.04
*Total Successful passes*	natural	25.48	9.20	26.28	7.76	28.63	10.37	19.22	5.58
	artificial	25.38	11.25	26.71	11.88	28.10	11.93	19.74	6.51
*Success rate of passes*	natural	0.67*	0.09	0.67*	0.09	0.69	0.09	0.66	0.10
	artificial	0.69	0.10	0.70	0.11	0.70	0.09	0.67	0.09
*Passes defensive third*	natural	6.53**	4.61	9.79**	3.94	6.05	4.07	2.40	2.04
	artificial	5.35	4.05	8.19	4.26	4.88	2.87	2.10	1.92
*Successful passes defensive third*	natural	5.81**	4.26	8.58**	3.80	5.50	3.93	2.15	1.82
	artificial	4.72	3.85	7.28	4.26	4.32	2.71	1.77	1.71
*Success rate of passes defensive third*	natural	0.88	0.14	0.86	0.10	0.89	0.15	0.90	0.18
	artificial	0.85	0.18	0.86	0.12	0.86	0.16	0.82	0.26
*Passes middle third*	natural	18.39	6.65	19.64	4.67	20.58	7.73	13.02	3.88
	artificial	17.88	8.21	19.42	7.96	20.11	9.07	12.64	3.94
*Successful passes middle third*	natural	13.67	5.57	13.74	4.41	15.81	6.53	10.15	3.28
	artificial	13.92	7.71	14.74	8.02	15.98	8.52	9.93	3.55
*Success rate of passes middle third*	natural	0.74*	0.10	0.69*	0.10	0.76	0.08	0.78	0.10
	artificial	0.76	0.11	0.72	0.13	0.78	0.09	0.78	0.09
*Passes final third*	natural	10.94	4.91	8.68	4.49	12.57	5.27	11.72	3.50
	artificial	10.76	5.27	8.18	4.99	12.03	4.94	12.53	4.74
*Successful passes final third*	natural	5.62	3.31	3.71	2.75	6.82	3.52	6.58	2.33
	artificial	6.30	3.87	4.37	3.65	7.28	3.65	7.58	3.41
*Success rate of passes final third*	natural	0.49**	0.16	0.39**	0.16	0.53**	0.14	0.56	0.12
	artificial	0.55	0.15	0.49	0.18	0.59	0.13	0.60	0.11
*Touches own half*	natural	31.55**	16.44	45.07**	9.49	30.16*	13.87	13.50	8.02
	artificial	27.32	14.72	39.92	10.91	25.83	10.49	12.13	7.00
*Touches opposition half*	natural	27.48	14.69	16.33	13.43	32.09*	11.92	36.82	9.10
	artificial	26.27	14.03	17.89	14.42	29.17	12.01	33.72	9.74
*Crosses*	natural	1.25	1.48	0.98	1.41	1.44	1.69	1.37	1.14
	artificial	1.41	1.46	1.20	1.51	1.47	1.53	1.63	1.27
*Successful Crosses*	natural	0.25	0.46	0.23	0.38	0.30	0.60	0.21	0.27
	artificial	0.31	0.48	0.27	0.46	0.34	0.49	0.33	0.51
*Dribbles*	natural	3.27**	2.74	1.66**	2.04	3.76**	2.46	4.92**	2.86
	artificial	1.75	1.75	0.88	0.98	1.61	1.32	3.14	2.21
*Successful dribbles*	natural	1.94**	1.73	1.10**	1.36	2.30**	1.60	2.62**	1.95
	artificial	0.67	0.77	0.37	0.46	0.65	0.63	1.11	1.05
*Success rate of dribbles*	natural	0.62**	0.26	0.67**	0.29	0.64**	0.23	0.53**	0.22
	artificial	0.40	0.29	0.49	0.35	0.39	0.26	0.35	0.25
*Tackles*	natural	3.06**	1.75	2.97**	1.73	3.64**	1.78	2.28**	1.39
	artificial	1.95	1.22	2.08	1.25	2.26	1.19	1.35	1.00
*Tackles won*	natural	2.35**	1.42	2.27*	1.38	2.80**	1.53	1.75**	1.03
	artificial	1.68	1.13	1.84	1.20	1.87	1.11	1.19	0.92
*Success rate of tackles*	natural	0.77**	0.20	0.76**	0.20	0.76*	0.19	0.77**	0.22
	artificial	0.85	0.20	0.88	0.20	0.81	0.20	0.89	0.18
*Clearances*	natural	1.94	2.30	3.94	2.45	1.07	1.33	0.35	0.41
	artificial	2.03	2.14	3.89	2.28	1.27	1.17	0.55	0.71
*Interceptions*	natural	2.40*	1.51	3.09	1.41	2.47*	1.46	1.23**	0.99
	artificial	2.10	1.60	3.24	1.43	2.02	1.34	0.65	0.66
*Fouls*	natural	0.99*	0.81	0.77	0.77	1.15	0.89	1.07	0.64
	artificial	1.18	0.90	0.88	0.76	1.36	1.01	1.35	0.82
*Yellow cards*	natural	0.09	0.18	0.10	0.16	0.11	0.22	0.06	0.12
	artificial	0.11	0.22	0.13	0.21	0.10	0.20	0.11	0.26
*Red cards*	natural	0.00	0.03	0.00	0.03	0.00	0.00	0.01	0.04
	artificial	0.00	0.03	0.01	0.05	0.00	0.00	0.00	0.00

* *p* <0.05

** *p* <0.01; M = Mean; SD = Standard Deviation.

Depending on the players' position, defenders on natural grass showed a higher number of passes in the defensive third, successful passes in the defensive third and touches in the own half (ES from 0.13 to 0.16); while on artificial turf they showed a higher rate of success in passes in the middle third, and passes in the final third (ES from 0.10 to 0.17). The midfielders on natural grass showed a higher number of shots, touches in their own and opposite half, and interceptions (ES from 0.10 to 0.12). On artificial turf, the midfielders showed a higher rate of success in passes in the final third (ES: 0.16). Finally, the strikers showed more interceptions on natural grass (ES: 0.15).

Nevertheless, in every players' position, we observed the same differences in tackles and dribbles. On natural grass, all of the player positions had a significantly higher number of dribbles and successful dribbles (ES from 0.12 to 0.37). On the other hand, on artificial turf, every player position showed a higher success rate of tackles (ES from 0.10 to 0.22).

Table 3 summarizes the discriminant analysis that identifies which variables allowed for better identification of the groups (natural grass or artificial turf). The models have a percentage of correctly classified cases greater than 85%, for both the total sample and for each of the playing positions. The variables that showed a higher level of discrimination among groups, including the entire sample and different game positions were: the number of passes in the defensive third, the number of passes in the middle third, the number of successful passes in the middle third, the success rate of passes in the middle field, the success rate of passes in the final third and the number of tackles.

**Table 3:** Discriminant analysis between field positions at the FIFA Women's World Cup 2011 and 2015.

	Total	Defenders	Midfielders	Strikers
Goals	–0.17	–0.24	–0.23	0.12
Shots	0.17	0.14	0.21	–0.06
Passes	0.50	–0.28	0.94	–2.57
Total successful passes	–0.81	0.14	–1.49	1.65
Success rate of passes	0.23	–0.08	0.33	0.47
Passes defensive third	0.42	1.42	–0.34	0.74
Successful passes defensive third	0.21	–1.14	0.89	–1.13
Success rate of passes defensive third	0.09	0.31	–0.13	–0.07
Passes middle third	3.52	3.26	3.04	–3.26
Successful passes middle third	–3.71	–3.87	–2.88	3.76
Success rate of passes middle third	0.80	0.86	0.69	–1.95
Passes final third	–0.09	0.24	–0.30	2.93
Successful passes final third	–0.09	-	0.59	–1.72
Succes rate of passes final third	–0.35	–0.37	–0.61	0.32
Touches own half	–0.51	–0.01	–0.61	1.27
Touches opposition half	0.50	0.22	0.03	–0.11
Crosses	–0.29	–0.28	–0.22	0.83
Successful crosses	-	0.11	-	-
Dribbles	0.17	–0.46	0.76	–0.02
Successful dribbles	0.38	0.82	–0.06	–0.80
Success rate of dribbles	0.23	0.06	0.43	0.04
Tackles	1.24	1.60	1.15	–0.38
Tackles won	–0.82	–1.31	–0.68	–0.27
Success rate of tackles	–0.11	–0.03	–0.04	0.47
Clearances	–0.03	–0.02	–0.06	0.26
Interceptions	0.09	–0.23	0.35	–0.49
Fouls	–0.26	–0.13	–0.32	0.23
Yellow cards	0.01	0.05	0.15	0.22
Red cards	0.17	0.22	-	–0.18
Eigenvalue	1.01	1.16	1.56	1.79
% Variance	100.00%	100.00%	100.00%	100.00%
Canonical correlation	0.71	0.73	0.78	0.80
Wilks Lambda	0.49	0.46	0.39	0.36
Chi square	289.54	93.11	153.95	101.45
Df	28.00	28.00	28.00	28.00
Correct classification	85.40%	88.30%	92.20%	89.60%

The variables that best discriminated between the surfaces in the total sample were: total passes, successful passes, touches in own and opposite half, successful dribbles and tackles won. In the case of the defenders, the discriminant factors were the number of successful passes in the defensive third, the number of dribbles, successful dribbles and tackles won. When we wanted to differentiate between the two World Cups, the midfielders also discriminated the following variables: total passes, successful passes, the success rate of passes, successful passes in the defensive third, successful passes in the final third, the success rate of passes in the final third, touches in the own half, crosses, tackles won, interceptions and fouls. Finally, the variables that best discriminated in the case of strikers were: total passes, successful passes, the success rate of passes, successful passes in the defensive third, passes in the final third, touches in the own half, crosses, successful dribbles, the success rate of the tackles and interceptions.

## Discussion

The artificial turf playing surface at FIFA Women's World Cup 2015 was a topic of discussion, with many voices against holding the tournament on this surface [30]. There were even players who refused to attend the event for this reason [31]. Thus, this study was the first to highlight the differences in the technical parameters of players in matches played on two different surfaces during two world competitions (natural grass during the FIFA Women's World Cup 2011 and artificial turf during the FIFA Women's World Cup 2015). This study showed a number of differences regarding technical actions of attack, confirming previous studies of elite male soccer [19, 32, 33]. No significant differences were found in the quantity of goals or shots made, which are fundamental key performance indicators, as elite soccer matches generally end with fewer goals scored [34].

On natural grass, we found a significantly higher number of passes were made, but the success rate of passes was higher on artificial turf. The uniformity of the surface is one of the most important parameters of artificial turf fields [24]. Since the vast majority of passes are made at ground in soccer, the excellent uniformity of the artificial turf fields plays a very important role in the success of the pass. A higher number of passes has been associated with the success of a team [19, 29, 35], however, a study of Alves et al. [36] showed that passing success, shots and shots on target are key performance indicators of winning teams. These results are in agreement with those of Liu et al. [17] who found that the winners at the 2014 FIFA World Cup demonstrated that shots, and shots on target, increased the chance of victory by 13% and 48%, respectively. On the other hand, Collet [37] noted that the key performance indicators of the success of a team during a competition, above the number of passes, were passing accuracy, shooting accuracy and passes-to-shot-on-goal ratio. During the FIFA Women's World Cup Canada 2015 in artificial turf fields, there was a higher percentage of successful passes in the midfield and offensive third (the most complex area for passing the ball). Near the opposite goal, there is a greater concentration of defensive players, with less space, and then the difficulty of the pass is greater. Soccer players indicate that a good passing accuracy requires a stable and uniform surface [38]. A better uniformity of the soccer field (as the artificial turf fields) benefits the success of the pass, especially in areas where the passing accuracy is essential.

According to the number of passes and successful passes by position, we found that both variables were highest for defenders; this may be due to the increased player density closer to opposition's goal [39, 40]. This fact highlights the importance of technical skills of defenders and midfielders in today's soccer, which aims to build possession from the back, through to the offensive areas of the pitch [18]. In this sense, Bush et al. [39] showed the changing role of central defenders in recent years, from a purely defensive role to making increasing offensive contributions. Another important aspect is the higher number of successful defensive actions (tackles and interceptions) carried out on natural grass versus artificial turf. These data have been highlighted in previous research [32, 38] and can be explained by the negative perception of skin abrasion perceived by players when they play on artificial turf [38, 41]. However, the latest research on women's soccer indicates that there is not an increased risk of injury on artificial turf compared to natural grass, contradicting previous research [42]. Another explanation could be the increased number of successful passes on artificial turf than natural grass, which would make retaining possession much easier [32]. The findings show that the playing style of teams may be slightly different on artificial turf (characterized by a more direct game, with fewer passes, but more precise and less aggressive defensive actions) than the playing style on natural grass. This may also explain the higher number of dribbles made on natural grass, including the ratio of total dribbles; a trend maintained by defenders, midfielders and strikers.

On the other hand, it is important to note than previous investigations similar physiological response on both surfaces [32, 43]. Furthermore, soccer on artificial turf do not induce higher fatigue or a delay in recovery [44]. In contrast, technical parameters in matches played on artificial turf evidenced a decrease in the incidences of slipping and an increase in the number of short passes [32]. The impact speed of the ball is another technical variable affected by the type of surface [45].

The main limitation of this study were factors that could not be controlled; the different number of players present in the two tournaments, and the players who made repeated appearances in both editions. Other data related to physical performance were not evaluated, as speed, movement patterns, playing time, or the exact recovery time of players. On the other hand, we did not consider other relevant variables as the travel distances of each player, the number of substitutions, or ball speed. There is another aspect that should be taking into account for future research, as the player’s previous load of training, that can vary depending on the competition level or the number of matches played during the season.

## Conclusions

This is the first article that analyses the influence of two different playing surfaces (natural and artificial) on the technical variables in elite soccer events (FIFA Women's World Cup). The key performance aspects were very similar between the two tournaments, the differences being found at a more discriminative level among tournaments, shown in passes, dribbles and tackles. However, we found more successful defensive actions have on natural than artificial turf. This result suggests that players are more comfortable tackling or carrying out aggressive defensive play actions on natural turf. Although artificial turf is a more sustainable choice in places with cold or dry climates, it can also be a good choice in places where natural grass is more popular, offering a game very similar to natural grass in most variables and generating a more direct and effective playing style in the same way.
